# The Prospective Non-Interventional DACCORD Study in the National COPD Registry in Germany: design and methods

**DOI:** 10.1186/1471-2466-15-2

**Published:** 2015-01-12

**Authors:** Peter Kardos, Claus Vogelmeier, Roland Buhl, Carl-Peter Criée, Heinrich Worth

**Affiliations:** Group Practice and Centre for Allergy, Respiratory and Sleep Medicine, Red Cross Maingau Hospital, Scheffelstrasse 33, 60318 Frankfurt am Main, Germany; Department of Respiratory Diseases, University of Marburg, 35043 Marburg, Germany; Pulmonary Department, Mainz University Hospital, 55131 Mainz, Germany; Department of Sleep and Respiratory Medicine, Evangelical Hospital Goettingen-Weende, 37120 Bovenden, Germany; Department of Pulmonology and Cardiology, Hospital Fuerth, University Erlangen-Nuernberg, 90766 Fuerth, Germany

**Keywords:** COPD, Non-interventional study, Out-patient, Pharmacological therapy, Register

## Abstract

**Background:**

A variety of large randomized controlled trials (RCT’s) evaluating pharmacotherapy in chronic obstructive pulmonary disease (COPD) patients does exist. One of the drugs that has been tested is the new long-acting anticholinergic glycopyrronium bromide.

**Methods:**

As the generalizability of results from RCT’s is questionable we designed a longitudinal, prospective non-interventional study (DACCORD) of two years duration plus two years extension with at least 6000 participants in approximately 500 primary and secondary care practices in Germany (within the new established COPD National Prospective Registry), to assess patient reported outcomes (PRO’s), lung function, adherence and drug safety. To circumvent the hurdle of inappropriate COPD diagnosis in a non-interventional trial, patients have to fulfill the inclusion criteria of the COPD disease management program (DMP) of the German statutory health insurances. Patient management should follow the German national COPD guidelines, which are based on Global Initiative for Chronic Obstructive Lung Disease 2007 (GOLD) report. Labels of prescribed drugs should also be taken into account. Patients received treatment as part of their standard care: at the discretion of the investigator patients were included in one of two arms. A: standard care with glycopyrronium containing regimen, and arm B: standard care without glycopyrronium.

**Discussion:**

For 2016 we expect important results regarding longitudinal development of PRO’s including exacerbations, lung function, adherence and side effects. We also investigate applicability of the new GOLD staging system in usual care. Data on diagnostic and treatment modalities in current German primary and secondary care, as well as pharmaco-economic data will be generated.

**Trial registration:**

1. German Register for non-interventional studies: http://www.vfa.de/de/arzneimittel-forschung/datenbanken-zu-arzneimitteln/nisdb.

2. EMA EnCePP http://www.encepp.eu/.

## Background

Chronic obstructive pulmonary disease is a major cause of morbidity and mortality worldwide; in Germany its overall prevalence in the population over 40 years is as high as 13.3% [1] and clearly increasing with age up to 40.4% in men older than 70 years. Thus, aging itself is a risk factor for COPD, continuing increase in prevalence in an ageing population is expected [2].

Since the early nineteen sixties COPD was recognized as an important health problem, even if initially different hypotheses and definitions applied: the Dutch hypothesis [3], as opposed to the British [4] until 2001 the Global Initiative for Obstructive Lung Disease (GOLD) defined COPD as “ airflow limitation that is not fully reversible … and usually …progressive” [5]. Data on the natural history of COPD, as measured by the annual rate of FEV_1_ (forced expiratory volume in 1 second) decline are scant [6] and controversial. A recent review found highly variable rates of decline but also increases in FEV_1_ in some patients [7]. There is evidence, that the decline is related to the baseline lung function [8, 9]. In the last decade it was increasingly recognized, that beyond FEV_1_ as a marker of disease severity other important variables determine the course of the disease: symptoms i.e. breathlessness, activity limitations, exacerbations [10] and, importantly co-morbidities [11]. However, to our best knowledge no data exist on the natural history of COPD under real life treatment conditions in the community (i.e. elderly patients, frequently with several co-morbidities likely with large impact on COPD outcome) so far.

Taking recent expansion of knowledge on the pathogenesis of COPD into account the 2011 version of the GOLD report introduced an entirely new and as yet controversial [12–16] complex system of assessing COPD based on symptoms, spirometry and exacerbation risk.

Therefore, we set up a large, nationwide COPD registry and at the same time initiated a prospective non-interventional study DACCORD of 2 years duration with optional further 2 years of extension. The study will primarily focus on patient related outcomes (PRO’s) in real life patients, treated in both primary and/or secondary care. Further assessments include exacerbations (frequency and time to first) and lung function variables. Beyond that, the design of the study will enable an evaluation either according to the old GOLD I-IV or the new GOLD ABCD grading system, which allows appropriate juxtaposition in the results.

According to both the old and new GOLD treatment recommendations long acting bronchodilators are the backbones of the treatment of COPD. In 2012 a new once-daily long-acting antimuscarinic (LAMA) glycopyrronium was introduced on the German market. The DACCORD study should also provide both post-authorization safety study (PASS) data for the European Medicine Agency (EMA) if needed and real life effectiveness data on pharmacologic treatment in accordance with guidelines.

## Methods

### Study design

DACCORD is an ongoing 2–4 yr non-interventional longitudinal prospective cohort study being conducted in 6000 patients at 500 centres in Germany. To further evaluate the therapeutic effects particularly of new and innovative medicinal products as part of standard COPD care in Germany, the study will consist of a group A: standard care with glycopyrronium containing regimen, and group B: standard care regimen without glycopyrronium. Randomization is not possible in a non-interventional study. Our overall target was however, to achieve an approximately 2:1 distribution. Each investigator was provided with an eCRF account to include patients in a 2:1 manner (group A: group B). However, after calling the CRO the investigator was able to modify this distribution. Having made a decision on any change in the treatment of an eligible COPD patient (s. below) the investigator included the patient in the respective study arm. This procedure enables collection of sufficient safety and real-life efficacy data of glycopyrronium. Thus, included patients in both arms were treated at the discretion of the physician, but following the German National Guideline NVL COPD (http://www.awmf.org/uploads/tx_szleitlinien/nvl-003l_S3_COPD_abgelaufen.pdf) and the label of the drugs prescribed.

Following a baseline visit, subjects are to be followed-up for at least 2 years, in approximately three months intervals. Thereafter 2 additional visits are planned at 3 and 4 years, respectively.

The study is being conducted in accordance with the Declaration of Helsinki, and has been approved by the ethics committee of the Friedrich-Alexander University Erlangen-Nuremberg.

### Study objectives

The main objective of the registry is the documentation, description and optimization of diagnostic and therapy of out-patient treated patients suffering from COPD in Germany. Further specific objectives will be evaluated:

1. To measure individual PRO’s i.e. dyspnea, symptoms, exacerbations by means of validated questionnaires CAT (COPD Assessment Test), mMRC (modified Medical Research Council scale), and weighted symptoms according to the new PRO questionnaire [17]).
2. To document exacerbations retrospectively before (6 month preceding inclusion) and prospectively (number, severity, treatment, time to next exacerbation) after inclusion.
3. To evaluate comorbidities (number, type, impact).
4. To analyze longitudinal changes in lung function including FEV_1_ decline.
5. To establish safety and tolerability of the treatments AE’s (adverse event), SAE’s (severe adverse event).
6. To assess the implementation of pharmacological treatment recommendations in German COPD guidelines at the participating centres.
7. To assess type of diagnostic measures performed during an outpatient visit.
8. To evaluate potential differences in the treatment of COPD between primary and secondary care.
9. To gather pharmaco-economic data.
10. To assess the potential impact of rapid onset of effect of glycopyrronium in the practical setting.
11. To assess patient adherence to pharmacological COPD treatments (as measured by refill rates).
12. To evaluate the dropout rate.

### Patients

All consecutive patients with COPD in participating centres should be considered for study enrolment in line with the inclusion/exclusion criteria if – at the discretion of the physician - they needed modification of their established antiobstructive treatment (Group A) or if a glycopyrronium-based therapy was started for treatment modification (Group B).

In order to guarantee the best possible selection under the conditions of a non-interventional study, only patients were eligible if they fulfilled recruitment criteria for DMP-COPD of the German statutory health insurance system. This strategy ensured, that – independently from investigator site – only spirometrically proven COPD patients were included in the multicentre real life study.

These eligibility criteria include:

- Age ≥ 40 years;
- Post bronchodilator FEV_1_/VC ratio <70%
- Change in FEV_1_ post bronchodilator – pre bronchodilator <15% or 200 ml (for reversibility also historical data were accepted).
- Alternatively: Static hyperinflation and increase in airway resistance measured by bodyplethysmography in patients with FEV1/VC (vital capacity) ratio >70%.

Moreover, doctor diagnosed COPD was also required. Despite DACCORD being a non-interventional study patients have to be willing and able to give informed consent for participation and pseudonymized study data collection.

Exclusion criteria are only ongoing participation in a randomized controlled trial and recruitment to DMP Asthma.

### Outcome measurements

At baseline (Visit 0) sociodemographic data (e.g. sex, year of birth, height and weight) and medical data e.g. physical exam, history including smoking status, exacerbations, vaccination status (influenza and pneumococcae), spirometry including reversibility test, the presence of co-morbidities, current COPD therapy including non-pharmacological treatment and concomitant non-COPD medication will be collected and registered electronically at the treatment site and stored in pseudonymised format in a secure, non-public database (see below). Furthermore each patient will be asked to complete the CAT, the mMRC and the new PRO symptoms questionnaire [17]. The collection of symptoms, measured by CAT and mMRC, history of exacerbations and spirometry assessment will allow the patients’ categorization according to the GOLD stages 2011, also in comparison with GOLD stages 2007.

**Figure 1:**
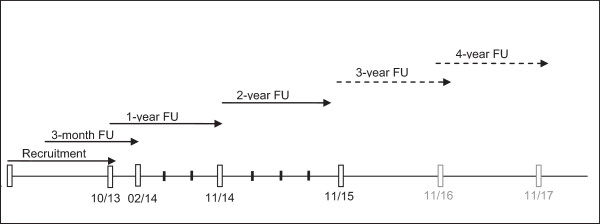
**DACCORD Study time table.**

At the next appointment after about three months according to the usual treatment interval in the COPD German Disease Management Program (visit 1), the outcome of the treatment modification or new treatment will be evaluated using the PRO questionnaire. Furthermore, changes in medication and exacerbations, hospital admissions and adverse events or serious adverse events and their outcome will be documented at each of the quarterly follow-up visits (visits 2, 3, 5–7). After one year (visit 4) and two years (visit 8), parameters will be documented in line with Visit 0. Further annual visits after three and four years, respectively, are optional (Figure 1).

While AE’s and SAE’s will be collected at each visit on standardized electronically forms, in case of adverse events of particular interest (cardiac events; e.g. ischaemic heart diseases, cardiac arrhythmias, cardiomyopathies and events associated with narrow-angle glaucoma), additional information concerning the event will be documented. After such events have occurred, corresponding questionnaires will be provided by Novartis Pharma GmbH – Department of Drug Safety on a case-by-case basis.

### Statistical considerations

#### Sample size calculation and population

There are approximately 930 respiratory physicians in Germany. At least 300 are likely to participate in this study, corresponding to about 33% of the above group and therefore ensuring representative coverage.

In order to make a comparative analysis of the approaches of different groups of doctors to the treatment of COPD, at least 100 general practitioners (specializing in pulmonology, defined as the possibility to conduct spirometry at the surgery) should additionally participate in the study. An average of 15 patients per centre are expected to be enrolled, producing a total sample size of 6 000 patients. The distribution of the centres and the planned number of patients will ensure a representative picture of outpatient treatment for COPD in Germany.

Furthermore, because the patient number is large, a number of relevant subsets can be identified and defined (e.g. older patients, women/men, patients with frequent exacerbations, patients with comorbidities, COPD stages, etc.).

#### Statistical analyses

In order to permit comparisons between the two study arms (with and without glycopyrronium, respectively), propensity score stratification will be performed. The main risk factors included therein are age, sex distribution, COPD severity, smoking status, number of exacerbations, AEs and concomitant diseases. The analyses will be differentiated, modeled on randomized clinical trials using the ITT (intend-to-treat) and PP (per-protocol, participants in accordance with all inclusion/exclusion criteria) population. All the parameters recorded will be analyzed descriptively. However, systematic group differences with respect to prognostic factors must be anticipated in a non-randomized study, leading to bias from confounding. Epidemiological analyses techniques to avoid confounding (e.g. propensity score matching or matched-pair analysis) might lead to selectively choosed patients and thereby additionally decrease sample size within certain groups. For these kind of analyses the credibility increases with the amount of patients enrolled. Under real-life-setting as in this study, the size of subgroups cannot be controlled but will correspond to the true distribution of these characteristics in the target population and thus might lead to small sample sizes in uncommon subgroups.

### Study organization

The study was designed and guided by the study steering committee consisting of a principal investigator and 4 respiratory physicians, working in both hospital and secondary care outpatient setting. Electronic data are collected at the study sites, data transfer, management and storage, quality control rest on the independent Lung Research Institute (ILF GmbH) of the German Respiratory Society. Statistical plan and evaluation will be provided by an independent service. The documentation of the data will be verified by on-site source-data verification at about 5% of the centres per year. The study is registered at http://www.vfa.de/de/arzneimittel-forschung/datenbanken-zu-arzneimitteln/nisdb.

DACCORD is fully sponsored by Novartis Pharma GmbH, Germany.

## Discussion

The non-interventional real life study design of DACCORD aims at bridging the gap between randomized controlled trials and clinical reality. Archibald Cochrane wrote: “Between measurements based on randomized controlled trials and benefit …in the community there is a gulf which has been much under-estimated” [18]. In fact, RCT’s are the most favorable way to assess therapeutic effect versus placebo or versus established treatment with high internal validity; however the external validity may be low. Thus, assessing the effect of a drug both RCT’s and natural studies including non-interventional studies are necessary [19, 20].

Hence, going beyond the results of randomized clinical trials with the non-interventional setting of DACCORD, the main strengths of the study are:

1. Size of the study population and long-term follow-up time period

Most epidemiological studies are cross-sectional by design or at best perform a pre-/post-analysis focusing on a particular question like guideline adherence [21]. Little data are available on repeated evaluations using identical methods and the same population obtained at a defined time point, although recent publications indicate the growing significance of data from observational studies [19, 22]. To our best knowledge no longitudinal studies in COPD patients with comparable size according to both the number of recruiting physicians and included COPD patients have been published, another similar study Canadian Cohort of Obstructive Lung Disease (CanCOLD) is just ongoing [22].

The longitudinal character allows for assessments of real life decline in lung function under usual care, quality of life changes, exacerbations, adherence and dropout.

2 Broad inclusion criteria

Most pivotal pharmacologic trials still exclude usual real life patients with COPD suffering from significant co-morbidities [23, 24]. Even after more than a decade of successful marketing realistic data of side effects of the most popular COPD treatments are not readily available, resulting in questionable estimations of - for example cardiovascular side effects [25, 26]. To our knowledge just one ongoing RCT is targeted to recruit and treat COPD patients with cardiovascular co-morbidity [27]. The natural character of the DACCORD study will expand currently available knowledge derived almost exclusively from controlled trials with narrow inclusion/exclusion criteria, performed in highly selected patients eliciting low external validity.

3. Implementation of DMP criteria as inclusion criterion

Large, post marketing non-interventional studies in respiratory medicine - partly conducted by family physicians - frequently suffer from inclusion of inappropriate patients not fulfilling the indication for the respective treatments and therefore leading to lower evidence of the results. In order to minimize potential risk of insensitive patient selection resulting in decrease of data quality, included patients need to fulfill the criteria of the disease management program (DMP) of COPD. Several DMP programs were implemented by the statutory health insurances in 2001 to further improve the treatment of patients with chronic diseases, e. g. diabetes or COPD. By performance of this inclusion criterion, all patients had had quality controlled appropriate lung function tests. All COPD patients with flow limitation and aged above 40 years are eligible for the DMP-COPD. However, some COPD patients with high (more than 15%) reversibility are at the time of the study recruitment not eligible for DMP-COPD, thus, not eligible for DACCORD. For patients treated in German surgeries this criterion ensures the external validity of the study.

Another important result of this study could be the comparison of the old and the new GOLD staging system applied in a large, well characterized prospective cohort. Will the complex GOLD ABCD system better predict patient related outcomes? Does real life medication fit better with the old or with the new staging system?

Both arms of the study will be analyzed statistically, as mentioned to generate data on real life effectiveness of glycopyrronium as monotherapy or combination treatment. Furthermore, a sample size of 6000 patients will allow performing adequately powered subgroup analyses on different pharmacological COPD treatment strategies.

Data for comparative effectiveness research will be also generated by means of analyses of accordance with guidelines and labels. Due to the high number of physicians and patients a reliable comparison between the two most important provider groups for respiratory diseases (family physicians and secondary respiratory care physicians) will be also possible.

If compared with register studies extracted from health insurance provider databases, the prospective character and the better identification and characterization of the target population, the inclusion of important but in insurance databases not readily available medical data, i.e. smoking habits, exacerbations, lung function is a great advantage.

A further strength of the study is the independent data management and statistical analysis by the Lung Research Institute founded by the German Respiratory Society on the one and the management of adverse events by the professional drug safety department of Novartis Germany on the other hand.

Patient enrolment started in November 2012; we await final results of main analyses in the first quarter 2016. Interim one year data will be available 2015.

In conclusion one of the largest ongoing prospective non-interventional studies could generate new, real life patient related, lung function and health care related data and answer important yet open questions to different aspects of COPD care.
